# Cross-talk between gut microbiota and liver steatosis: Complications and therapeutic target

**DOI:** 10.1515/biol-2022-0699

**Published:** 2023-08-30

**Authors:** Yuan Yao, Yunfeng Shen

**Affiliations:** Department of Metabolism and Endocrinology, The Second Affiliated Hospital of Nanchang University, Nanchang, China; The Queen Mary School, Nanchang University, Nanchang, Jiangxi 330031, China; Department of Endocrinology and Metabolism, Institute for the Study of Endocrinology and Metabolism in Jiangxi Province, The Second Affiliated Hospital of Nanchang University, 330006, Nanchang, China

**Keywords:** gut microbiota, fatty liver steatosis, complications, therapeutic markers, literature review

## Abstract

Liver steatosis is the most widespread chronic liver condition. Its global incidence is rising swiftly and is currently estimated to be 24%. Liver steatosis is strongly related with numerous metabolic syndrome characteristics, like obesity, insulin resistance, hyperlipidemia, and hypertension. The gastrointestinal tract contains about 100 trillion commensal organisms and more than 7,000 distinct bacterial strains. Fat deposition in the liver without secondary causes is known as liver steatosis. Dysregulation of the gut flora is one of the factors connected to the onset of fatty liver disease. Dietary choices may alter constitution of the microbiome and cause gut microbiome dysbiosis, particularly due to the intake of food high in fructose sugars, animal products, and saturated fats. Various gut bacteria cause nutrient metabolism in multiple ways, setting off different inflammatory cascades that encourage liver disease and pathways that help fat build up in the liver. Due to their relatively stable nature, genetic factors may not be responsible for the constant increase in liver steatosis incidence. Genetic factors set the stage for liver steatosis pathogenesis. This review will offer an overview of our present knowledge of the roles played by gut microbiota in regulating the development of liver steatosis, potential side effects, and potential treatment targets.

## 1 Introduction

The term “non-alcoholic fatty liver disease” (NAFLD) refers to several ailments caused by deposition of fat in the liver. Obese or overweight people frequently exhibit it. Early-stage NAFLD is typically not dangerous; however, if it progresses, it can cause significant liver injury, like cirrhosis. High-fat levels in the liver are also related to an upsurge likelihood of substantial health problems such as high blood pressure, diabetes, and renal ailment. NAFLD enhances a person’s probability of developing cardiac issues if they already have diabetes [1]. NAFLD etiology involves a complicated combination of environmental factors (i.e., Western diet), obesity, alterations in microbiota, and predisposing genetic variations, as a result, lipid homeostasis is disturbed, and triglycerides and other lipid species accumulate excessively in hepatocytes. Insulin resistance (IR) is a fundamental mechanism that causes endoplasmic reticulum stress, lipotoxicity, disrupted autophagy, and, eventually, hepatocyte damage and mortality, which causes inflammation in the liver, stellate cell stimulation, and progressive fibrogenesis, promoting the development of the ailment [2]. NAFLD typically manifests as part of the metabolic syndrome (MetS) and is intimately linked with dyslipidemia, IR, obesity, and type 2 diabetes mellitus (T2DM). Patients with NAFLD have a greater overall mortality rate than the rest of the population, primarily related to T2DM and cardiovascular risk factors. Persons with non-alcoholic steatohepatitis (NASH) also have higher liver-related mortality rates because cirrhosis, fibrosis, and hepatocellular carcinoma (HCC) are more likely to develop. The complex pathophysiology of NAFLD is still not completely understood. The precise role of environmental and genetic factors and extrahepatic and intrahepatic events in defining the disease phenotype is still uncertain, despite latest developments in our understanding of the molecular and cellular mechanisms underpinning disease genesis and progression [3].

Metabolic-associated fatty liver disease (MAFLD), formerly known as NAFLD, has developed as the main cause of liver illness globally. MAFLD is becoming increasingly costly, clinically and economically, as the global obesity pandemic spreads. The term MAFLD refers to all fatty liver disease states, which corresponds to the conventional understanding that NAFLD signifies a range of liver illness related to IR, initiating with pure “benign” steatosis (NAFL) and progressing to NASH. This inflammatory state can result in advanced fibrosis or cirrhosis [4].

Liver steatosis is a minimum of 5% without common factors contributing to secondary hepatic fat accumulation, for instance, chronic viral hepatitis, congenital hepatic disorders, excessive alcohol consumption, autoimmune hepatitis, or prolonged usage of steatosis-inducing drugs. Cardiometabolic illness, which includes liver steatosis, is frequently but not always accompanied by obesity, T2DM, hypertension, and dyslipidemia. An estimated 25.24% of individuals globally suffer from liver steatosis, having the greatest incidence in South America (30.45%) and the Middle East (31.79%) [1]. Asia, North America, Europe, and Africa have a respective rate of 27.37, 24.13, 23.71, and 13.48%. According to reports, there are between 20 and 50 occurrences of liver steatosis per 1,000 person-years in various nations. Such alarming figures render liver steatosis a significant clinical and financial burden, becoming the most recent global chronic liver disease epidemic. Potentially, liver steatosis may transform into cirrhosis, fibrosis, and hepatocellular cancer [1,5].

There are numerous microbial communities in the digestive system of humans. They promote metabolism, digestion, and absorption and are a biological barrier in a symbiotic interaction. Animal and human research have shown the potential pathogenicity of intestinal microbial diseases in developing NAFLD/NASH. As a crucial organ for nutrition absorption, the digestive tract develops an excellent barrier network to prevent the absorption of hazardous substances. Proper function of the intestinal barrier is essential for retaining the intestinal environment’s equilibrium [4]. The composition of the various barriers varies, but they are all closely related. Biological and chemical intestinal barriers have been described. Intestinal bacteria encourage intestinal epithelial cells to generate a variety of immune mediators, including cytokines and chemokines, which are essential for maintaining the integrity of the epithelial barrier, developing the mucosal immune system, and controlling the host immune response [6]. In conjunction with the mucosal immune system, it influences the development of its effector function antigen recognition, recruitment, and proliferation. It can also affect the host from a distance by the metabolites influx from the gastrointestinal tract (GIT) into the systemic circulation and via modulation of migrating immune cells, which might considerably impact distant tissues. The metabolites and bacterial constituents that pass through the epithelial barrier go into the bloodstream and can potentially have a bioactive influence throughout the body. For instance, the liver and bacteria of the host body break down the l-carnitine present in red meat to create trimethylamine-*N*-oxide (TMAO), which encourages atherosclerosis. When given the same amount of l-carnitine, bacterial colonies from vegans and vegetarians make less TMAO than those from omnivore humans [7].

Additionally, the microbiota has significant involvement in shifting the ratio of pro- to anti-inflammatory signals, which subsequently contributes to inflammation and may advance to liver steatosis. Thus, there is a pressing need to comprehend the pathogenesis of liver steatosis. Doing so may help in bringing about improvement in patient stratification, diagnostics along with the finding of new therapeutic targets. The following sections will discuss the methods through which the gut microbiota both favorably and unfavorably influence the onset and course of liver steatosis.

## 2 Pathogenesis

Identifying the underlying causes of NAFLD and NAFLD-related fibrosis is essential for the discovery of diagnostic biomarkers and the identification of treatment targets; therefore, we begin with a discussion of the most essential features of NAFLD-related fibrogenesis.

### 2.1 From liver steatosis to NASH

One of the most important elements in NAFLD formation is gain of weight, which is often the result of a sedentary lifestyle characterized by a high-calorie diet and reduced physical activity. Having a high caloric intake and minimal consumption of energy, the liver plays a key role in sustaining the metabolic equilibrium. The accumulation of lipids, as observed in NAFLD, is a significant factor in the formation of lipotoxicity. The formation of chronic inflammation, fibrosis, and oxidative stress are accelerated by lipotoxicity. In addition, dietary carbohydrates (particularly fructose) are assimilated by the liver and transformed to free fatty acids (FFA) through the process of de novo lipogenesis. Approximately 40% of the liver lipids are derived from dietary carbohydrates and fat. The remaining 60% are made up of malfunctioning adipose tissue. Consequently, IR causes an upsurge in FFA flux, which has a deleterious impact on the liver. The FFA are ordinarily degraded by beta-oxidation in the mitochondria. The mitochondria are overburdened by an excess of FFAs, leading to mitochondrial uncoupling. They generate reactive oxygen species as a result. This, in conjunction with adipose tissue abnormalities and endotoxins from the intestines, causes NASH [8]. Factors such as Kupffer cells, immune cells, and liver macrophages infiltrating the liver lead to NASH’s inflammatory condition. Large quantities of FFA are absorbed by Kupffer cells, which lead them toward an inflammatory phenotype. This results in the release of inflammatory cytokines comprising tumor necrosis factor (TNF), interleukin (IL)-6, and IL-10. IL-6 and TNF are both linked to the development of NASH [9]. NASH is the result of a complex interaction between multiple factors, such as genetic alteration and adiposity, which creates a profibrotic environment in the liver [9].

### 2.2 From NASH to liver fibrosis

In the case of chronic liver illness such as NAFLD, immune responses not only restore tissue function but also cause tissue damage. A hyperactive or excessive immunological response might lead to organ malfunction and concurrent fibrotic tissue deposition and cell loss [9]. These immune responses include adaptive and innate components, i.e., neutrophil infiltration is frequently observed in NASH patient’s histologic specimens. In addition, individuals with NASH and NASH-related advanced fibrosis have a greater ratio of neutrophils to lymphocytes than patients without NASH [10].

Immune cell infiltration stimulates the transdifferentiation of hematopoietic stem cells (HSCs) into collagen-producing myofibroblasts. Typically, this procedure is involved in short-term tissue restoration. In response to liver injury, HSCs get activated and differentiate from the dormant phenotype into propagative and contractile myofibroblasts. HSCs both store and produce retinoids glial fibrillary acidic protein (GFAP) during their quiescent phase. When stimulated, retinoids and GFAP are gradually lost, which corresponds with the formation of myofibroblasts and the production of extracellular matrix (ECM) components like type I, type III, and type IV collagen, along with hyaluronic acid (HA) [11]. HA glycosaminoglycan polymer levels are proportional to the severity of liver fibrosis. The development of collagen is followed by a rise in ECM-degrading metalloproteinases (MMPs), i.e., MMP-9. The collaboration of active and excessively expressed MMP-9 with type III collagen deposition leads to an excess of cleaved type III collagen items, like plasma N-terminal propeptide of type III procollagen or neo-epitope PRO-C3 [9].

During the differentiation, the HSCs’ characteristic star-like morphology transforms into a droplet-like form. The procedure is subsequently balanced by anti-fibrotic mechanisms, leading to myofibroblast deactivation or apoptosis and the resolution of the scar. In chronic illnesses such as NAFLD, these processes are out of balance. The imbalance will result in continuous stimulation of propagating, contractile, as well as migratory fibroblasts. This results in an inordinate amount of ECM production. The abundance of ECM will demolish the liver’s physiological architecture. Non-parenchymal cells (NPCs) such as Kupffer cells and other immune cells, which are brought to the location by the demise and hepatocyte apoptosis, regulate this equilibrium. NPCs will begin to generate pro-fibrogenic cytokines [12].

## 3 Liver steatosis and microbiota of the GIT

The human gastrointestinal system is populated by at least 10^14^ distinct types of microorganisms, with anaerobic bacteria accounting for the majority, including 500–1,000 species. These commensal gut bacteria often serve the host well, assisting with general metabolism (such as bile acids, BAs) and the transformation of food into nutrients and energy (for instance, short-chain fatty acids, SCFAs) [13].

Studies on mice and fecal transplantation trials have revealed that the microbiota of the GIT has a causal role in the emergence of liver steatosis. First, cohousing studies with healthy wild-type mice and mice predisposed to NASH because of genetic alterations in the inflammasome pathway show that coprophagia – the sharing of microbiota – causes wild-type mice to develop inflammation and liver steatosis. In addition, several NAFLD changes are replicated by direct fecal microbiota transplantation (FMT) (from germ-free recipients to weight-matched obese mice with or without steatosis). The degree of hepatic gene expression implicated in lipid absorption, fatty acid catabolism, lipogenesis, and VLDL export is raised, along with the hepatic triglyceride content and content in the liver as a whole [14]. Weight-matched mice with or without steatosis showed different gut microbiota compositions, with steatotic mice showing a rise in two bacterial species (a relative of *Barnesiella intestinihominis* and *Lachnospiraceae* bacterium 609). These behaviors were linked to these changes. Though mouse models can study liver disease and associated microbiota, there are several limits to extending results to humans from rodent trials. Models based on mice do not acquire the full range of histological abnormalities seen in human liver steatosis (i.e., hepatocyte ballooning or cirrhosis), and the illness is not always linked to obesity and IR, such as in the case of human liver steatosis, as detailed in a lengthy review [15]. Although some rodent models (choline-deficient mice, for example) may eventually develop identical ultimate histological changes as those detected in humans, the pathophysiology is entirely different between humans and mice because the latter typically experience weight loss [15].

Furthermore, there are significant differences between the microbiotas of humans and mice concerning the constitution (the great majority of genera present in mice are not there in humans), pre-dominant genera, and the prevalence of certain genera and species. Finally, there are important variations in the digestive tract architecture of humans and mice that also influence the overall makeup of the GIT microbiota [16]. Due to these restrictions, it is difficult to assess how the gut microbiota contributes to liver steatosis in mouse models. By means of FMT from sick patients to germ-free mice to mimic the patient’s hepatic phenotype is one way to overcome this obstacle. FMT between steatosis-affected people and germ-free mice transfers several liver steatosis features, including inflammation and liver steatosis, exacerbated by a high-fat diet (HFD) [16].

Nevertheless, germ-free mice possess underdeveloped immune systems [17], and the development of metabolic illnesses depends on inflammation and/or a balance of the immune system. Since traditional animals have a more advanced immune system and permit donor microbiota engraftment, using traditional mouse models for FMT investigation may be an alternate technique to examine the function of the microbiota in models of rodent [17]. Particularly, within 14 days, Chow diet-fed conventional mice after receiving feces from obese women with liver steatosis have increased hepatic lipid content. Aside from a few of these restrictions, data from rodent-based investigations support the notion that the typical microbiota of the GIT has a key involvement in the emergence of liver steatosis [18]. The pathways via which the gut microbiome may participate in NAFLD formation and its evolution to NASH have been the subject of several hypotheses. In a nutshell, these include the action of various metabolites produced by microbes (such as ethanol, choline, or TMAO) and BA signaling, which may additionally influence immunity, as well as a raised intestinal permeability resulting in the release of lipopolysaccharide (LPS) to the host, causing tissue and systemic inflammation. Based on these ideas, investigations involving humans have examined the gut microbiota makeup of individuals with liver steatosis and a healthy liver as controls in order to find microbiota or microbiome-related metabolite signatures capable of being employed as a noninvasive tool for diagnosis [19]. This review now focus on the microbiome profiles seen in steatosis and liver steatosis in humans (mostly adults, though we also reviewed some pediatric research). Notably, obesity and T2DM are associated with intestinal dysbiosis. We talk about signatures that are present in those metabolic illnesses as well.

**Figure 1:**
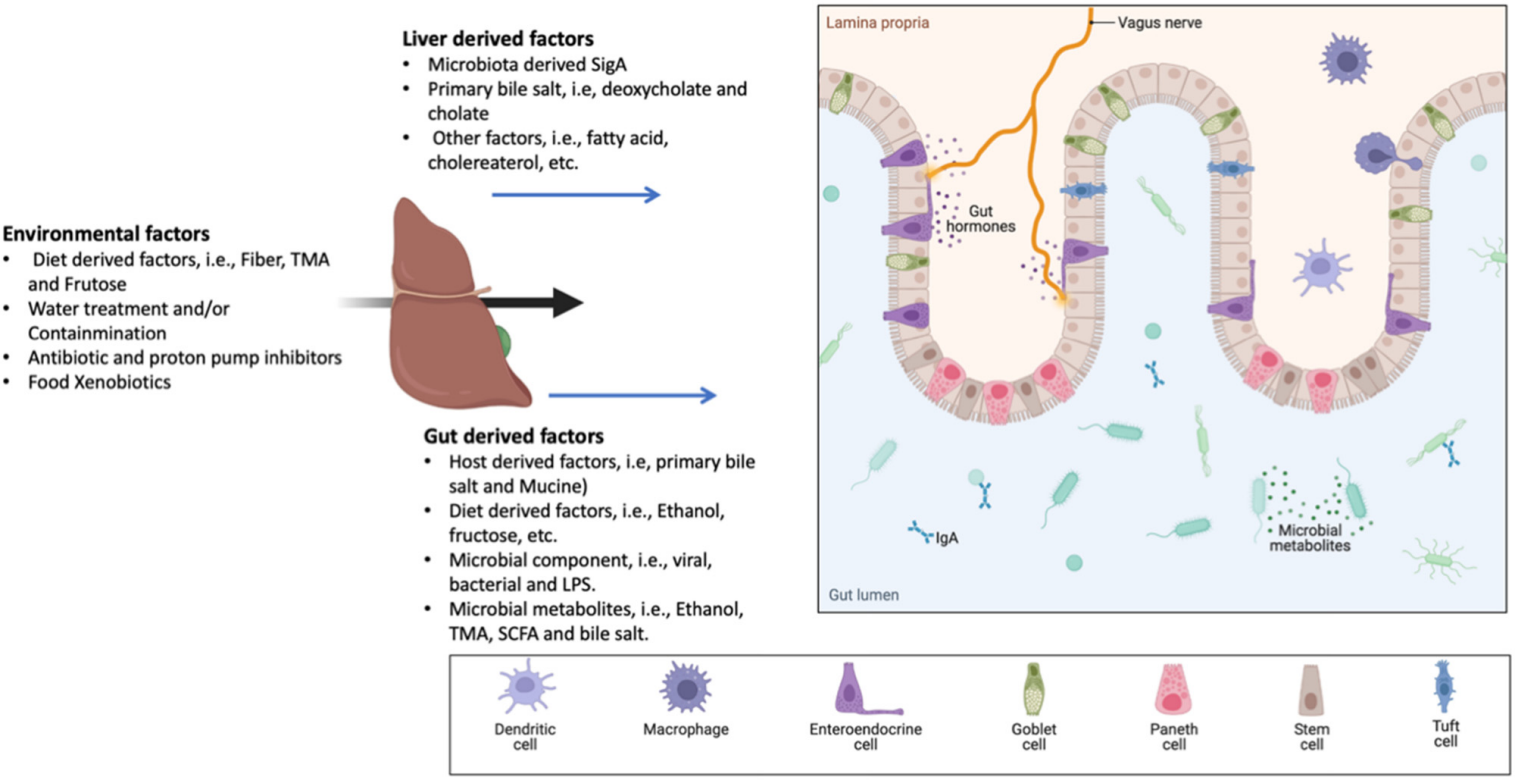
The connection between the gut and liver and the function of the gut microbiota in NAFLD development. Liver steatosis is brought on by the changed gut flora caused by environmental factors. The malfunctioning liver’s inability to restore intestinal eubiosis creates a vicious cycle that accelerates the formation of NAFLD. There is no evidence of communication through systemic mediators. Microbe-associated molecular pattern (MAMP), NAFLD, SCFA, deoxycholic acid (DCA), and chenodeoxycholic acid (CDCA), LPS, and trimethylamine (TMA) are all examples of fatty acid.

Along with systemic circulation, the biliary tract and portal vein play a role in the bidirectional communication of the gut-liver axis. Liver-derived components like BAs influence the constitution and function of the microbiota within the GIT. Together, the liver’s glucose and lipid metabolism are controlled by GIT-derived substances that may be nutritional or microbial. When environmental variables such as gut dysbiosis and/or increased intestinal permeability disrupt the gut-liver axis, the liver experiences pro-inflammatory alterations. The liver’s inability to control the gut microbiota results in additional disease development (Figure 1). It is essential to have a thorough understanding of gut-liver communication to create effective strategies for diagnosis, therapy, and prevention.

## 4 NAFLD preventive measures and current treatment strategies

Losing weight and reducing metabolic risk factors are currently the gold standard for treating NAFLD. According to data, dietary intervention is the most efficient NAFLD and NASH therapy. Even a 5% weight loss can help with steatosis, and a 10% weight loss can help with steatohepatitis. Nevertheless, several patients struggle to maintain the suggested lifestyle changes. Several interventions that could benefit NAFLD management have been investigated. NAFLD therapy options have included weight reduction pharmaceuticals such as metformin, anti-oxidant vitamins E and C, thiazolidinedione (TZD), ursodeoxycholic acid (UDCA), polyunsaturated fatty acids (PUFA), and lipid-lowering medications. However, most trials have been brief and have not included histology endpoints [20].

In the 96-week PIVENS trial, which included patients with non-diabetic NASH, vitamin E was compared against pioglitazone, a medicine for diabetics. Both therapies significantly reduced liver steatosis and lobular inflammation. However, neither produced a meaningful impact on the scores of hepatic fibrosis. Despite the observed inability to see an influence upon hepatic fibrosis scores, guidelines advise administering 800 IU/day of vitamin E in non-diabetic patients with NASH (confirmed from biopsy) as appropriate. However, in diabetic patients, vitamin E should not be used as a routine treatment for NASH or NAFLD without liver biopsy, cryptogenic cirrhosis, or NASH cirrhosis until more evidence supports its efficacy. To accurately evaluate the effectiveness of the available NASH therapy options, larger randomized controlled studies with histological outcomes are required [21].

Gut microbiota integrity depends at least partly on a diet. In comparison to “animal-based meals” made up of cheese, eggs, and meats, “plant-based diets” abundant in fruits, vegetables, legumes, and grains have shown a major impact on the makeup of the gut flora. An animal-based diet encourages the growth of the gut microbiota, increasing the synthesis of branched-chain fatty acids and deoxycholic acid, a precursor to BAs. Additionally, people who consume an animal-based diet have a higher degree of expression of the genes for the breakdown of polycyclic aromatic hydrocarbons (cancer-causing substances), vitamin production, and b-lactamase. Saturated fat and fructose have a higher chance of promoting hepatic lipid buildup and the development of NASH. A high-fructose diet in mice increases gut-derived portal endotoxemia, which, by activating toll-like receptor 4 (TLR4) and TNF-α, causes liver steatosis [7]. Hence, recommendations involving the promotion of diets low in fructose and saturated fats may offer protection against NAFLD development.

Probiotics (commensal bacteria in pill form) and prebiotics (substances that stimulate the growth or activity of “good bacteria”), which, given as supplements over the counter, have been suggested as potential treatment strategies for gut microbiome dysbiosis. In studies using animal models, Lactobacillus has received the most attention among the products currently on the market. A decline in liver steatosis was observed in diet-induced obese mice following 8 weeks of oral *Lactobacillus rhamnosus* administration, supported by liver biopsies [22]. Additional investigations in human subjects involving Lactobacillus are therefore warranted.

**Figure 2:**
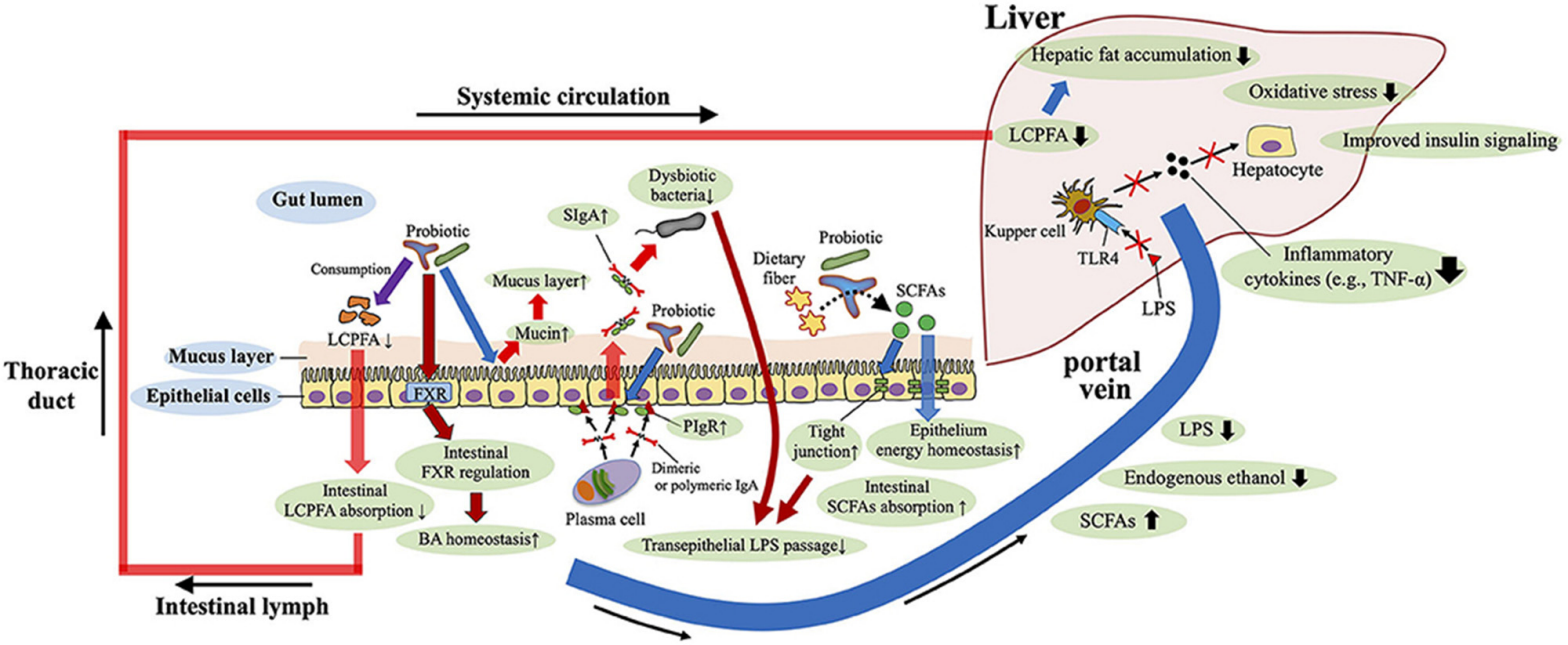
Mechanisms of dysbiosis-induced NAFLD in children and adolescents. Dysbiosis and disruption of the gut microbiota contribute to the development of non-alcoholic fatty liver disease (NAFLD) via modulation of the gut–liver homeostasis, including the involvement of the gut barrier, bacterial endotoxin [lipopolysaccharide (LPS)], endogenous ethanol, bile acids (BAs), and short-chain fatty acids (SCFAs). FGFR4, fibroblast growth factor receptor 4; FXR, farnesoid X receptor; TGR5, G-protein-coupled bile acid receptor; TLR4, toll-like receptor 4; TNF-α, tumor necrosis factor-α; UDCA, ursodeoxycholic acid [24].

Given that gut dysbiosis has a key function in the pathomechanisms of NAFLD, it seems sensible to consider using probiotics and fecal transplantation as therapies that target the gut microbiota. Numerous research in mice and people have established the value of probiotic supplementation in preventing NAFLD. Probiotics have anti-fibrotic properties, and *Lactobacillus rhamnosus* GG supplementation reduces the amount of BA-induced liver damage and fibrosis in bile duct-ligated animals by boosting intestine farnesoid X receptor (FXR)-mediated inhibition of BA production and increasing BA excretion [23]. Furthermore, mice were prevented from developing high-fructose diet-induced NAFLD utilizing *Lactobacillus rhamnosus* GG supplementation, thereby boosting good bacteria, lowering portal LPS transfer, and re-establishing the function of the gut barrier (Figure 2).

According to a recent study, *Lactobacillus rhamnosus* GG administered orally inhibits the growth of NAFLD in mice fed with a HFD by ingesting intestinal fatty acids, thus decreasing their absorption [24] (Figure 2). The administration of mice with IgA-coated *Lactobacillus jensenii* (as opposed to supplementation with IgA-free bacteria) substantially reduced HFD-induced dyslipidemia and disruption of the gut mucosal barrier. Additionally, administration of IgA-coated *Lactobacillus jensenii* increased colonic butyrate synthesis, mucin-2, and polymeric Ig receptor mRNA expression, all of which improve the gut’s barrier function (e.g., IgA secretory levels, mucus layer, and tight junction tension) [24] (Figure 2). The right probiotic strain must be chosen to reap the benefits of probiotics.

### 4.1 Conventional NAFLD treatment techniques and their interactions with gut microbiota

There is currently no validated therapy for NAFLD in pediatric patients who are obese. However, weight loss focused on dietary alterations and augmented exercise levels are often the main NAFLD therapeutic techniques in adults and obese pediatric patients. Transient elastography shows that in adolescents and obese NAFLD children, weight loss based on diet and activity interventions is beneficial in lowering hepatic fat deposition [25]. Although the exact method by which food and exercise modify the gastrointestinal microbiota to treat NAFLD is unknown, it is undeniably advantageous to maintain a healthy gut microbiota through a proper diet and regular exercise. Regarding exercise, athletes’ gastrointestinal microbiome diversity is higher than healthy non-athletes. Without regard to diet, aerobic exercise raises fecal concentrations of SCFAs by changing the diversity of the gastrointestinal microbiota in adult humans [25]. Exercise shields mice from an environmental toxin’s alteration of the gut microbiota.

Additionally, exercise training over 12 weeks improved the deleterious microbiome profile associated with obesity and decreased microbial inflammatory signaling in obese children. Similarly, several proteins working with complex-valued polynomial model may also be implemented to benefit the conventional therapeutic approaches [26,27]. Chinese obese children with simple diet-induced simple obesity or Prader-Willi syndrome experienced considerable weight loss and altered dysbiotic gut microbiota profiles due to dietary intervention that included a non-digestible carbohydrates-based diet [28]. Together, these findings imply that gut microbiota is indispensable in the exercise and diet exercise-mediated improvement of NAFLD in adolescents and children. Presently, no pharmacological cure for NAFLD has been approved by the Food and Drug Administration, but a few treatments are being investigated with promising outcomes (Table 1).

**Table 1:** Current NAFLD therapeutic possibilities [12]

Procedure	Drug/treatment option
Surgical and weight-loss drugs	Orlistat and Bariatric surgery
Phlebotomy	—
Pharmacologic intervention	Anti-TNF-α agents, UDCA, Vitamin E, betaine, silymarin, statins, fibrates, N-3 PUFA, metformin, TZDs, and incretin-based therapies.
Lifestyle modifications	Exercise and calorie restriction

As an anti-NAFLD therapeutic nutrient, Vitamin E is widely given as a supplement to children and adults, combined with exercise and dietary modification. According to a meta-analysis, vitamin E improves adult NAFLD patients’ histological and biochemical features, particularly NASH. A double-blind, placebo-controlled, randomized trial involving children with NASH indicated that vitamin E treatment dramatically improved histologic results but did not lower ALT levels. In a different pediatric trial, vitamin E and hydroxytyrosol reduced the systemic inflammation associated with NAFLD, mostly by raising blood IL-10 concentrations in reaction to DNA damage recovery, reducing steatosis and hypertriglyceridemia [29].

Although there is no direct proof that vitamin E supplementation improves NAFLD by reducing gut microbiota dysbiosis, numerous studies show that vitamin E supplementation positively affects the gut microbiota [30,31]. Vitamin E has improved gastrointestinal microbiota disturbed by colitis and protects intestinal barrier function in the dextran sulfate sodium-induced colitis mouse model [30]. The development of NAFLD is stimulated by intestinal barrier disruption and an upsurge in portal LPS produced by dysbiotic gut flora. Therefore, the impact of vitamin E on the diversity of the intestinal barrier and gastrointestinal microbiota might point to the existence of gut microbiota-mediated mechanisms for the improvement of NAFLD brought on by vitamin E supplementation [30].

### 4.2 Pharmacological intervention

The etiology of NAFLD is diverse and complex, making diagnosis challenging. Consequently, it is difficult to treat patients with NAFLD at various stages successfully. As a result, several personalized therapies that focus on NAFLD at a specific stage are advised individually [32]. To treat progressive NASH (fibrosis stage 2), pharmacological medication therapy is suggested by the EASL-EASD-EASO Clinical Practice Guidelines. Individuals with initial-stage NASH who also have diabetes, MetS, or enhanced liver function must as well be included in pharmacological drug treatment since they have a significant chance of the illness progressing. NAFLD pathophysiological pharmacological therapeutics are being developed, but the shortage of authorized drug therapy is due to response rates. These pharmacological therapies used to treat fibrosis seem to have a minor impact. Despite extensive clinical research, there are no FDA-approved NASH treatments, and no specific course of treatment is advised. The medicines now being prescribed for NASH are utilized off-label in every country [33].

### 4.3 Microbiota-based intervention

Scientists worldwide are looking for alternative techniques to treat NAFLD following decades of rigorous pharmacological interventions to find tailored medications for the condition. Probiotics are used as microbial therapeutics in these therapies. Several preclinical and clinical investigations have been conducted after observing the impact of gut microbiota on effectively preventing and treating NAFLD, NASH, and NAFLD-HCC. However, several probiotics, including Pediococcus, Bifidobacterium, and Lactobacillus, have shown promise in reversing NAFLD in preclinical animals. By re-establishing microbial balance in the gut, which decreases lipogenesis and ultimately lowers liver inflammation, probiotic therapy in rodents eliminates NAFLD [34]. The positive effect of probiotics in human disease conditions has been optimized and translated through several clinical trials over the past 10 years; however, microbiota-based medicine is still in the early stages of research.

Sharpton et al. discovered the positive association of probiotics or synbiotics with significant improvement in liver function enzymes, liver stiffness measurements, and liver steatosis (which together reflect NASH conditions) in a recent meta-data analysis and systematic review based on 21 randomized controlled trials that summarized microbiota-based targeted therapeutic strategies (9 probiotics and 12 synbiotics) in NAFLD patients. Given the population’s diversity, the range of phenotypes of liver illness, and the lengths of probiotic or symbiotic therapies, they may have positive effects that are particular to the liver [35].

Antibiotics, prebiotics, pre- and probiotic combinations (synbiotics), and FMT are further approaches that aim to positively change gut microbial assemblages to impact NAFLD. Prebiotics are dietary fibers that are indigestible but fermentable and are specifically used by the gastrointestinal microbiota to benefit the host microbiota. Prebiotics include short- and long-chain β-fructans, fructooligosaccharides (FOS), inulin, lactulose, and galactooligosaccharides. These are the most extensively studied prebiotics for chronic liver diseases. The administration of lactulose decreases the likelihood of recurrent HE and HE-related hospitalization and lengthens life expectancy by controlling symptomatic hyperammonemia in cirrhosis with HE patients [36]. Liver function enzymes, lipid profiles, and inflammatory markers have been shown to have positively influenced synbiotic supplementation, as revealed by a meta-data analysis of RCTs in NAFLD patients [37] (Table 2).

**Table 2:** Additional prospective NAFLD therapies and their intended targets

Therapeutic options/drugs	Target	References
Probiotics (VSL#3)	Bacterial overgrowth	[38]
Cytoprotective agents (i.e., UDCA)	Apoptosis	[39]
Novel treatments (i.e., incretin analogs, oligofructose, and angiotensin-converting enzyme inhibitors/blockers of angiotensin receptors)		
Anti-TNF agents (pentoxifylline)	Pro-inflammatory cytokines	[40]
Vitamin E, beta-carotene, silymarin, lecithin, N-acetyl-cysteine, betaine, and other vitamins	Oxidative stress	[36]
Omega-3 fatty acids, statins, and fibrates	Dyslipidemia	[41]
Meglitinides, metformin, and TZDs	IR	[37]

Despite the positive elements of synbiotics, an investigation recently found that individuals with NAFLD who used synbiotics (FOS with Bifidobacterium animalis subspecies lactis BB-12) only saw a change in their gut microbiome without any reduction in steatosis or fibrosis [42]. As opposed to placebo medication, another clinical investigation found that liver steatosis and fibrosis were significantly reduced by symbiotic supplementation in lean NAFLD patients. Compared to the placebo group, lipid profiles, and several inflammatory indicators dramatically decreased [43]. Both investigations imply that important metabolic dysfunctional elements may prevent the resolution of hepatic damage markers. Additional research is required to ascertain whether the study’s main result affected the metabolic factors.

## 5 Liver steatosis and potential complications

The gut microbiota can play a key role in the onset and the advancement of NAFLD to cirrhosis and carcinoma of the liver. Growing research reveals that gut bacteria and its metabolites have a direct influence on intestinal architecture and immune response, leading in aberrant inflammatory stimulation and intestinal endotoxemia; gut dysbiosis also causes gut-liver axis dysfunction via changes in the BA metabolism pathway [44]. Few therapies are useful in the cure and prevention of NAFLD. Pioglitazone might benefit people with advanced NASH or T2DM, although credible clinical evidence is insufficient. The word “drug-resistant” refers to the ability of a substance to withstand the effects of drug use. Statins can lower serum LDL cholesterol concentrations and avoid cardiovascular problems; however, they do not address the development of a liver disorder. By modifying or reshaping the gut microbiota by means of antibiotics, probiotics, synbiotics, prebiotics, and FMT to preserve gut homeostasis, MTT is a novel approach for treating NAFLD. Commercialized strains of Streptococcus, Lactobacillus, and Bifidobacterium enhance the inflammatory milieu in the gut, encourage the survival and development of intestinal epithelial cells, and suppress pathogenic bacteria via modifying host defense and the immune system [45].

A potential therapeutic approach for developing anti-NAFLD drugs may involve using a particular bacteria or bacterial compound to interfere with NASH. By colonizing the gut with *Akkermansia muciniphila*, metabolic problems brought on by HFD may be reversed, oxidative stress reduced, inflammation inhibited, liver function improved, and the gut microbiota normalized. Colesevelam, a BA sequestrant, disrupts the hepatic-intestinal circulation of BA and has been demonstrated to impact cholesterol, lipid, glucose homeostasis, decreasing hepatic fat accumulation in NASH patients [46].

NASH frequently develops from NAFLD, particularly in people with T2DM. Lobular inflammation, hepatocellular necrosis, and frequent fibrosis are characteristics of NASH. Numerous investigations have now demonstrated that patients with fibrosis and NASH experience the highest mortality rate. Cirrhosis develops as fibrosis worsens. Cirrhosis development varies greatly in terms of its rate. It is influenced by factors like age, BMI, type 2 diabetes, blood pressure control, and the severity of steatohepatitis. Obesity (visceral obesity or increased excessive BMI), T2DM, and the development of moderate to extreme fibrosis are the three most important risk factors [47]. However, the exact causes of cirrhosis are still unknown, given the significant degree of heterogeneity in disease progression.

A traditional drug having immunomodulatory, anti-oxidant, and anti-apoptotic properties is UDCA. Clinical investigations have not yet verified its impact on NAFLD/NASH. However, several UDCA derivatives, including nor-UDCA, presently undergoing phase II clinical trials, have demonstrated positive therapeutic effects in mouse NASH models. These studies indicated that dysfunctional gut microbiota could contribute to NAFLD’s onset, development, and even worsening carcinogenesis [48]. It has become crucial for NAFLD management and treatment to understand how to make advantage of gut microbiome change or inhibit the evolution of NASH as well as identify bacteria and products from bacteria as potential targets. First, while novel approaches like prebiotics, probiotics, phage therapy, FMT, and FXR/TGR5 agonists have showed potential in the cure of NAFLD, the difficulties in producing therapeutic medications that are both safe and effective, such as tissue specificity, selectivity, as well as medication resistance for long-term usage need to be investigated further.

### 5.1 NAFLD phenotypes

Several studies have attempted to build diagnostic models incorporating metabolomics, such as lipidomics, with or without other laboratory or clinical characteristics. The goals of such models were primarily to determine NAFLD phenotypes, for example, the existence or lack of fibrosis, and to differentiate between NAFL and NASH [49]. Several studies have focused on noninvasive methods for assessing the phenotype of NAFLD or anticipating hepatic fibrosis, which may minimize the need for biopsies in NAFLD patients at risk for NASH. Research on the metabolomics toward the formation of metabolites-based noninvasive characterization of NAFLD have been expedited in the last 10 years by knowledge of the pathogenetic process and the advancement of high-throughput technology. There is currently no broadly accepted metabolomics marker for NAFLD phenotype or severity, even though numerous intriguing biomarker candidates from various latest research. The following are some hypotheses as to why accurate and precise markers are few. First, NAFLD is a multisystem disease that involves a complicated interaction of gut microbiota, concomitant metabolic conditions, lack of physical exercise, and lifestyle risk factors, include bad food habits and the absence of physical exercise [50].

The discovery of metabolomics biomarkers for particular phenotypes will help to advance personalized therapy in the area of NAFLD. Knowledge of the underlying mechanisms in the progression of NASH to NAFL and identifying metabolite biomarkers involved in NASH pathogenesis may cause the development of innovative diagnostic and treatment alternatives.

### 5.2 NAFLD and cardiovascular disease (CVD)

CVD affects patients with NAFLD and T2DM for various reasons, for instance, greater IR and intrahepatic lipid buildup. This is connected to a decline in the clearance of triglyceride-rich lipoproteins and an increase in hepatic VLDL secretion in the peripheral circulation. As a result, a proatherogenic profile is created, comprising an increase in tiny, dense LDL particles, inflammation, hypertriglyceridemia, and low HDL-C [51].

Additionally, these individuals frequently have extreme hepatic IR, worsening glycemic control over time. Hyperinsulinemia resulting from increased insulin production and reduced insulin clearance is associated with hepatic IR [52]. In epidemiological research and animal disease models, hyperinsulinemia has been linked to atherogenesis. In short-term human clinical investigations, chronic hyperinsulinemia has also resulted in acquired IR and downregulation of insulin signaling pathways. In this situation, hyperglycemia is more severe and might also be a factor in CVD [53]. Individuals with NAFLD have an elevated risk of cardiovascular ailment because of endothelial dysfunction. Independent of other risk factors, people with well-controlled T2DM and NAFLD have been found to have early left ventricular “diastolic dysfunction,” also known as HFpEF or heart failure with preserved ejection fraction [53].

Compared to clinically matched individuals without NAFLD, people with NAFLD frequently have an increased atherosclerotic disease and substantially poorer carotid intima-media thickness. Studies have linked this to rising levels of inflammation, steatosis, and/or fibrosis. With cirrhosis, CVD is the main cause of death in NASH [54]. Additional risk factors frequently cluster with NAFLD and CVD. T2DM, hypertension, hyperlipidemia, inflammation, and obesity, in addition to hyperinsulinemia and IR are the most critical risk factors.

### 5.3 NAFLD with chronic kidney disease (CKD)

The prevalence of NASH and NAFLD with fibrosis has lately been linked to CKD. Advanced-stage CKD relates to rather severe fatty liver disease. Most investigations have used increased albuminuria/proteinuria or an estimated glomerular filtration rate (eGFR) of less than 60 ml/min/1.73 m^2^ to define CKD [55]. In case-control research it was discovered that the degree of eGFR decline was independently correlated with the severity of the liver histology in individuals with biopsy-proven NASH [56].

An increased frequency of CKD was found among Japanese patients having biopsy-proven NAFLD, and the liver histology also deteriorated. Overall, they discovered that 14% of NAFLD patients had CKD. Only 6% of patients suffering from NAFLD with no indication of NASH had CKD, compared to 21% of patients with biopsy-proven NASH [57]. Compared to those without NAFLD or NASH, this was higher. Although the etiology of this connection is unclear, NAFLD’s enhanced atherogenicity is probably a significant component. A recent meta-analysis also revealed that CKD had a greater incidence in individuals with NASH compared to individuals with NAFLD without NASH. Patients with advanced fibrosis had a greater prevalence of CKD than individuals with lower fibrosis [58].

### 5.4 NAFLD and polycystic ovarian syndrome (PCOS)

Depending on the diagnostic criteria employed, PCOS affects between 5 and 18% of premenopausal women. It is distinguished by hyperandrogenism and ovulatory failure. Before diagnosing PCOS, it is necessary to rule out other diseases with the same symptoms. In the 1980s, it was discovered that IR was a key component of the syndrome long after its first description, affecting both obese and lean patients [59]. NAFLD is more frequent in PCOS, which has been categorized as a metabolic and reproductive illness due to the crucial role that IR plays in the pathogenesis of the condition. IR and obesity are the two primary risk factors for NAFLD in PCOS [59].

NAFLD has been observed to become more widespread in women with PCOS, which is more persisted when the patients have MetS risk variables such as T2DM, BMI, and hypertension. In women with PCOS, evidence of hyperandrogenism, particularly when testosterone levels are above 3 nmol/L, has been linked to a higher risk of NAFL [60].

### 5.5 Development of liver fibrosis

The evidence for liver fibrosis is also limited to *in vivo* and *in vitro* investigation and is scarce in this area. Apoptosis of hepatocytes with the release of a wide range of cytokines (i.e., interleukins [−1, −2, −18], hedgehog ligands, TGF-β, TNF-α, and numerous others) has been the focus of potential pathways connected to the development of NASH [61]. One such route (the transcriptional activator TAZ) was identified by Wang et al. that plays a key role in NASH, as demonstrated in a mouse model. At the same time, this complex signaling network, activated by damaged hepatocytes, causes the surrounding Kupffer cells to become active, which causes hepatic stellate cells to develop into myofibroblasts and upsurge the synthesis of matrix proteins, eventually leading to cirrhosis. Genetics appears to be involved because the PNPLA3-I148M mutation in NASH may directly affect stellate cell activity and alter lipid droplet metabolism with a liver-targeted GalNAc3-conjugated antisense oligonucleotide. A large decrease in liver inflammation and fibrosis was observed in PNPLA3 knock-in 148M/M mutant mice (having a human PNPLA3 I148M variant) [62].

On a clinical level, a recent study investigated variables linked to illness development in a significant clinical trial (*n* = 475 patients) [63]. The key factors related to clinical disease development are the degree of fibrosis at baseline and larger increments in the amount of hepatic collagen, enhanced liver fibrosis score, and increased alpha-smooth muscle actin level with time. In patients with bridging fibrosis (F3), progression occurred in 22% of cases over a 96-week follow-up period, whereas individuals with cirrhosis experienced clinical events linked to the liver in 19% of cases. Beyond liver histology, clinicians must acknowledge that T2DM and obesity is main risk factors for advancing liver ailment, necessitating screening and early management.

## 6 NAFLD prevention and treatment based on gastrointestinal microbiome

Since gastrointestinal dysbiosis plays a role in the pathomechanisms regulating NAFLD, it seems sensible to think about using probiotics and fecal transplantation as therapies targeting the gut microbiota. Numerous research in mice and people have established the value of probiotic supplementation in preventing NAFLD. Probiotics have anti-fibrotic properties, and *Lactobacillus rhamnosus* GG supplementation reduces the amount of BA by boosting intestine FXR-mediated inhibition of BA production and improving BA-induced liver damage, BA excretion, and fibrosis in animals with bile duct ligation [23]. Furthermore, *Lactobacillus rhamnosus* GG supplementation prevented mice from developing high-fructose diet-induced NAFLD by boosting good bacteria, re-establishing the role of the gut barriers, and lowering portal LPS transfer. According to an investigation conducted recently, *Lactobacillus rhamnosus* GG administered orally inhibits the formation of NAFLD in mice fed with HFD by limiting intestinal fatty acid absorption through the consumption of intestinal fatty acids. The administration of mice with IgA-coated *Lactobacillus jensenii* (as opposed to supplementation with IgA-free bacteria) substantially reduced HFD-induced disruption to the gastrointestinal mucosal barrier and dyslipidemia. Additionally, administration of IgA-coated *Lactobacillus jensenii* increased colonic butyrate synthesis, mucin-2, and polymeric Ig receptor mRNA expression, improving the gut’s barrier function (e.g., IgA secretory levels, mucus layer, and tight junction tension) [64]. The appropriate probiotic strain must be chosen, though, to reap the advantages of probiotics.

By lowering the levels of liver TLR4 and serum LPS, probiotics supplements may minimize liver disease, enhance gut microbiota structure, and reduce LPS-TLR4 signaling, slowing down the progress of NAFL. A supplement comprising probiotic bacteria 14 strains, given for 8 weeks, resulted in a significant decrease in fatty liver indices, levels of inflammatory cytokines (IL-6 and TNF-α), and aminotransferase activity in adult humans with NAFLD and T2DM [65]. Serum adipocyte hormones (resistin and leptin) were lowered in rats following probiotics supplementation combination, such as *Bifidobacterium bifidum, Lactobacillus acidophilus*, *and Lactobacillus plantarum*, thus moderating high-sucrose and HFD-induced steatosis. Pro-inflammatory signals were additionally found to be suppressed. A probiotic supplement comprising *Lactobacillus rhamnosus, Bifidobacterium bifidum, Lactobacillus acidophilus*, and *Bifidobacterium lactis*, administered for a total of 12 weeks, improved hepatic ultrasonographic findings and numerous biochemical factors (such as triglycerides, aspartate aminotransferase, and ALT) in obese Iranian children. Fibrosis, liver fat, and gut microbiota did not significantly change due to the probiotic treatment in another trial on obese Latino teenagers; rather, the probiotic enhanced obesity [66]. These outcomes recommend that probiotics may be helpful in treating pediatric NAFLD, but they also point out that the right bacterial strain must be chosen to reap the benefits.

## 7 Metagenomics: a route to comprehending the gut microbiome

The gastrointestinal microbiome is a significant factor of host health, but it has only been possible to study it at the genomic level since the development of next-generation sequencing in the last two decades. The sequencing of shotguns is beginning to shed light on the eukaryotic, prokaryotic, and viral elements of the gut population, exposing their taxonomy as well as the functions contained in their metagenome. This revolution in comprehension is being spurred by the ongoing advancement of sequencing technology, necessitating the growth of computational methods that is adaptable to the ever-changing nature of sequence datasets.

In addition to identifying the human diversity of gastrointestinal microbiome, metagenomics can potentially lead to the discovery of novel genes, microbial routes, and functional dysbiosis. Huge potential exists for the implementation of metagenomics to reveal the processes and links between the human microbiome of the intestines and illnesses. Though, there are limitations to metagenomics, which must be addressed [67].

With the rapid advancement and use of metagenomics, in addition to metabolomics, metaproteomics, and metatranscriptomics, it is feasible to discover new microbiological diagnostic markers for early detection and innovative therapeutics. Increasing the number of probiotics and maximizing the contribution of microorganisms are also very promising. On the basis of a greater awareness of the human microbiome’s role in illness and its relationships, interindividual variations, as well as physiological factors, personalized medicine research will advance significantly. Based on a comprehensive knowledge of ARGs in the gastrointestinal microbiome, it is also conceivable to investigate novel antibiotics that target microbiomes with resistance to antibiotics [68].

Recent metagenomic investigations of the human gut microbiota have been undertaken in small cohorts; therefore, it is critical to improve our understanding of the human gut microbiome by studying people from multiple countries, over longer time periods, including various age groups [69], and at different stages of illness. An investigation on the properties of the human gut microbiome at various phases of disease could help us comprehend the linking between gut microbiome and illness progression, thereby facilitating the development of the most effective approaches for preventing, treating, and even reverting disease.

Due to the limitations of metagenomics, it is essential to integrate other microbiome methods, such as cultivation methodologies, with a metagenomics investigation of the intestinal microbiome. This will guarantee more correct and undoubted results. In recent times, a number of investigations have utilized this combination successfully and achieved significant results. To surmount the limitations of metagenomics, it is also necessary to make a standardized technique for the extraction of microbial DNA, to enhance computational algorithms, and to finish references to databases [70].

The use of metagenomic technologies to the microbiome of the human digestive tract is still in its early stages. Though, it has been used in many different situations, such as soil and the ocean, for a considerable amount of time. Following the success of employing metagenomic technology to the study of these environments, further research can be conducted on the human intestinal microbiome. Along with bacteria, the human intestine also contains eukaryotes and viruses. Several research on eukaryotes and viruses utilizing the metagenomics method has been conducted to date; therefore, the prospective investigation of the human gastrointestinal microbiome utilizing the metagenomics technique appears promising, and further research are required immediately.

## 8 Conclusion

In conclusion, a substantial body of research points on the gut microbiota is essential for the etiology of liver steatosis. Environmental variables, particularly nutrition, and drugs, are the main disruptors of the gut microbiota. Due to their relative stability, genetic factors may not be responsible for the constant rise in liver steatosis incidence, but they set the stage for liver steatosis pathogenesis. Clinical investigations have revealed promising microbiome signatures linked to liver steatosis that may be exploited for noninvasive diagnosis or disease progression monitoring. However, additional investigation is required to check the usage of the signatures in large cohort longitudinal studies with good design that account for significant confounding factors, including comorbidities, such as T2DM and obesity, medication, food, and ethnic background. Combining microbial profiles with microbiota-derived compounds found in urine, feces, or blood/plasma may help improve these markers’ diagnostic and prognostic usefulness. With this method, liver steatosis subtypes may be differentiated more precisely, and the effectiveness of the treatment can be assessed with greater accuracy.

Moreover, efficient microbiome-based therapeutics can be developed if we thoroughly grasp the interactions between the microbiota and the liver. Two primary strategies can essentially be combined. The first may directly manipulate the gut microbiota through the elimination of undesirable strains, the introduction of beneficial strains (preferentially isolated from the normal microbiota of the human gut), or the FMT of the dysbiotic microbiota. Utilizing metabolites generated by microbes via stimulation or inhibition of their production will comprise the second strategy. Overall, the feasibility of an ideal study design is still up for debate, but it should be considered when discussing large-scale research consortiums. Another restriction is the natural hesitation of medical professionals, patients, and ethical committees to repeat liver biopsies. Finally, this sector requires sophisticated techniques for predicting microbiome signatures (such as multi-omics strategies combined with deep learning). Investigating the entire microbial ecology is crucial because interactions between microbes may be just as significant as the presence of many different families, genera, or species.

Similarly, it is important to consider the significance of microbiome-altering factors like viruses, phages, and fungi. Diagnosing individuals with liver abnormalities through routine treatment might be possible by combining microbiome profiles with systemic metabolites obtained from microbes. Future NAFLD-NASH therapeutic strategies will change the signatures to produce biomarkers, permitting essential follow-up for therapeutic response. However, it depends on the likelihood of developing reliable biomarkers. An in-depth understanding of the interplay between the liver and microbiota is expected to help us develop efficient therapies incorporating microbiota, such as FMT, metabolite manipulations, probiotic interventions, and noninvasive prognostic and diagnostic techniques which combine microbiota signatures and metabolites specific to the liver steatosis.
